# Probiotic Supplementation Facilitates Recovery of 6-OHDA-Induced Motor Deficit via Improving Mitochondrial Function and Energy Metabolism

**DOI:** 10.3389/fnagi.2021.668775

**Published:** 2021-05-07

**Authors:** Bira Arumndari Nurrahma, Shu-Ping Tsao, Chieh-Hsi Wu, Tu-Hsueh Yeh, Pei-Shan Hsieh, Binar Panunggal, Hui-Yu Huang

**Affiliations:** ^1^Graduate Institute of Metabolism and Obesity Sciences, College of Nutrition, Taipei Medical University, Taipei City, Taiwan; ^2^Ph.D. Program in Drug Discovery and Development Industry, College of Pharmacy, Taipei Medical University, Taipei City, Taiwan; ^3^School of Pharmacy, College of Pharmacy, Taipei Medical University, Taipei City, Taiwan; ^4^Department of Neurology, Taipei Medical University Hospital, Taipei City, Taiwan; ^5^Department of Neurology, College of Medicine and Taipei Neuroscience Institute, Taipei Medical University, Taipei City, Taiwan; ^6^Bioflag Biotech Co., Ltd., Tainan City, Taiwan; ^7^School of Nutrition and Health Sciences, College of Nutrition, Taipei Medical University, Taipei City, Taiwan; ^8^Department of Nutrition Science, Faculty of Medicine, Diponegoro University, Central Java, Indonesia

**Keywords:** Parkinson’s disease, 6-hydroxydopamine, probiotic, prebiotic, *Lactobacillus salivarius* AP-32, mitochondrial function, energy metabolism

## Abstract

Parkinson’s disease (PD) is a neurodegenerative disease associated with progressive impairment of motor and non-motor functions in aging people. Overwhelming evidence indicate that mitochondrial dysfunction is a central factor in PD pathophysiology, which impairs energy metabolism. While, several other studies have shown probiotic supplementations to improve host energy metabolism, alleviate the disease progression, prevent gut microbiota dysbiosis and alter commensal bacterial metabolites. But, whether probiotic and/or prebiotic supplementation can affect energy metabolism and cause the impediment of PD progression remains poorly characterized. Therefore, we investigated 8-weeks supplementation effects of probiotic [*Lactobacillus salivarius* subsp. *salicinius* AP-32 (AP-32)], residual medium (RM) obtained from the AP-32 culture medium, and combination of AP-32 and RM (A-RM) on unilateral 6-hydroxydopamine (6-OHDA)-induced PD rats. We found that AP-32, RM and A-RM supplementation induced neuroprotective effects on dopaminergic neurons along with improved motor functions in PD rats. These effects were accompanied by significant increases in mitochondrial activities in the brain and muscle, antioxidative enzymes level in serum, and altered SCFAs profile in fecal samples. Importantly, the AP-32 supplement restored muscle mass along with improved motor function in PD rats, and produced the best results among the supplements. Our results demonstrate that probiotic AP-32 and A-RM supplementations can recover energy metabolism via increasing SCFAs producing and mitochondria function. This restoring of mitochondrial function in the brain and muscles with improved energy metabolism might additionally be potentiated by ROS suppression by the elevated generation of antioxidants, and which finally leads to facilitated recovery of 6-OHDA-induced motor deficit. Taken together, this work demonstrates that probiotic AP-32 supplementation could be a potential candidate for alternate treatment strategy to avert PD progression.

## Introduction

The Parkinson’s disease (PD) is a widespread neurodegenerative disease in aging individuals, marked by a gradual loss of dopaminergic neurons in the SNc pars compacta (SNc) region of the midbrain. The gradual loss of dopaminergic neurons reduces dopamine turnover in the motor circuit of the brain leading to motor dysfunctions, such as bradykinesia, rigidity, tremor, and postural. Simultaneously, non-motor impairments, such as gut dysfunction, sleep disorder, depression, and dysphagia, are also associated with PD instability (Kalia and Lang, 2015; Maiti et al., 2017). PD patients also suffer from loss of body weight and skeletal muscles (Garber and Friedman, 2003; Falvo et al., 2008; Stevens-Lapsley et al., 2012), which all together worsen the life quality of PD individuals over time.

Although PD pathogenesis remains unclear, several risk factors are known to be associated with PD such as aging, environmental factors, and genetic mutations (Kalia and Lang, 2015; Maiti et al., 2017). Importantly, mitochondrial function is known to be disrupted in PD patients (Schapira et al., 1990). This disruption leads to decreased respiratory enzyme activity, decreased ATP production and energy failure (Verstreken et al., 2005). In a functional mitochondrion, a large antioxidant defense capacity balances the generation of reactive oxygen species (ROS). Mitochondrial damage with decrease of antioxidant defense capacity is imperative of net ROS production. Functionally compromised mitochondria, as in the state of PD, creates an imbalance between ROS production and removal, resulting in net ROS production along with detrimental effects on the anti-oxidative defense system. In effect, there is an increase in oxidative stress resulting from the increased ROS production, and accumulation with simultaneous decrease in oxidative stress-related antioxidant enzymes. Prolonged oxidative stress can further exacerbate mitochondria damage and eventually lead to dopaminergic neurons degeneration (Federico et al., 2012; Subramaniam and Chesselet, 2013; Tait and Green, 2013). An increase in glycolysis can counteract energy failure and dopaminergic cell death induced by malfunctioning mitochondria (Smith et al., 2018). However, neurons have limited capability to up regulate glycolysis. Augmenting the ATP deficit by stimulating glycolysis with pharmacological interventions has shown to attenuate PD progression (Cai et al., 2019).

Several recent studies show a link between PD pathogenesis and gut microbiota dysbiosis (Sun and Shen, 2018). Compared to healthy people, the gut microbiota of individuals with PD have been consistently reported to be abundant with more pro-inflammatory bacteria and less abundant with anti-inflammatory bacteria (Unger et al., 2016). Such changes in the abundance and diversity of microbial flora result in altered production of microbe-derived metabolites in the GI tract. Microbial metabolites beneficial to host immune system and energy metabolism, particularly the short-chain fatty acids (SCFAs), are found to be reduced in PD patients (Unger et al., 2016). The SCFAs (acetate, propionate, and butyrate) are used by host as substrates for lipid, cholesterol, and glucose metabolism which are eventually utilized for the production of energy via Krebs cycle in mitochondria (LeBlanc et al., 2017). Therefore, SCFAs play an important role as precursors for energy metabolism in host. This understanding has led to an ongoing hypothesis that gut microbiota could play an important role in PD pathogenesis, where SCFAs could be the key mediator between gut microbiota and brain bidirectional axis (Ferrante et al., 2003; Monti et al., 2010; Koh et al., 2016; Lei et al., 2016; Schonfeld and Wojtczak, 2016).

Probiotics are live microorganisms that, when administered in appropriate amounts, confer a beneficial effect on the host (Gazerani, 2019). Whereas, prebiotics are non-digestible nutrients that can influence gut microbiota growth and activity, including the production of SCFAs (Franco-Robles and López, 2015). *Lactobacillus*, is a popular probiotic known to exhibit neuroprotective effects (Castelli et al., 2020; Hsieh et al., 2020), decrease neuroinflammation, increase synaptic plasticity, alter gut microbiota (Bonfili et al., 2017), immunomodulatory effects, barrier function promoter and pathogen inhibitor functions (Hsieh et al., 2013). Besides probiotics, studies have also shown that the metabolites produced by the bacteria fermentation process could generate similar therapeutic effects (Koh et al., 2016). The residues obtained from probiotics culture medium are an alternate source of microbe-derived metabolites, which may contain beneficial nutrients, such as SCFAs. Therefore, the residual medium from probiotic culture can alternatively be utilized as prebiotic supplements.

In this study, we tested the hypothesis that probiotic and potential prebiotic from probiotic residual medium might perform a beneficial role in preventing neurodegeneration in PD condition, we also try to investigate and explore the possible mechanism underlying beneficial effects of a probiotic [*Lactobacillus salivarius* subsp. *salicinius* AP-32 (AP-32)], a potential prebiotic [AP-32 residual medium (RM)], and a symbiotic [the combination between AP-32 and its residual medium (A-RM)] supplementation in 6-OHDA PD rat model.

## Materials and Methods

### Probiotic and Prebiotic

Probiotic *Lactobacillus salivarius* subsp. *salicinius* AP-32 (AP-32) and the prebiotic residues of microbial culture medium (RM) were provided by Bioflag Biotech Co., Ltd., Tainan, Taiwan. *L. salivarius* subsp. *salicinius* AP-32 was isolated from healthy human gut and deposited in Food Industry Research and Development Institute, Taiwan (ID: BCRC 910437) and in Wuhan University, China (ID: CCTCC-M2011127). Probiotic AP-32 consists of 10^11^ colony-forming unit (CFU)/g. Probiotic AP-32 was selected due to its capacity to regulate inflammatory function and genus *Lactobacillus* function to promote neuroprotective effects (Bonfili et al., 2017; Castelli et al., 2020; Hsieh et al., 2020). RM powder was derived from the supernatant obtained from probiotic AP-32 fermented medium. Briefly, after the probiotic AP-32 were grown in the medium, the fermented medium was centrifuged to remove the bacteria. The supernatant was spray dried. 4 L of supernatant liquid RM upon spray-drying yielded 60 g of RM powders. The RM consists of carbohydrates, proteins, fats, amino acids, and SCFAs (Supplementary Table 1). A-RM was a mixture of probiotic AP-32 (1.03 × 10^9^ CFU/kgBW of AP-32) and RM (62 mg/kgBW of RM). Fresh stock solution of AP-32, RM, and A-RM were prepared before oral gavage everyday by dissolving each powder in distilled water to final concentration of 1 mg/ml. The final gavage volume was calculated as per the body weight of rats.

### Animal Grouping

**FIGURE 1:**
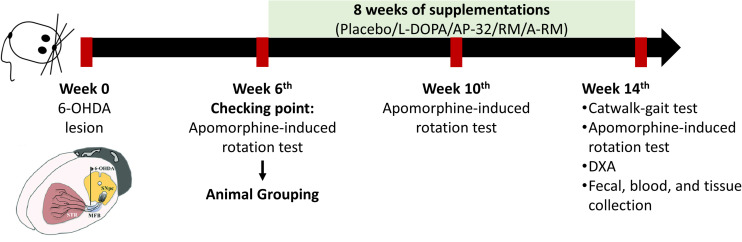
Timeline of the study.

Male Sprague-Dawley (SD) rats (8 weeks old, 280-300 g) were purchased from BioLASCO Taiwan Co., Ltd. (Taiwan) and were housed two rats per cage at Laboratory Animal Center (LAC) Taipei Medical University under a controlled environment (12/12-h light/dark cycle, 22°C–24°C, 40%–60% humidity) with free access to food and water. Rats were randomly assigned to normal control (NC, *n* = 5) and PD groups (*n* = 30). PD rats were obtained by unilateral 6-OHDA injection into the right medial forebrain bundle (MFB). To reduce variability among groups, we performed apomorphine-induced rotation test at 6 weeks after unilateral 6-OHDA lesions and confirmed 25 PD rats (contralateral rotation more than 100 rotations/hour (Kaminska et al., 2017)). The confirmed PD rats were equally assigned into the following 5 groups: untreated PD (PD, *n* = 5), L-DOPA-treated PD (L, *n* = 5), AP-32-treated PD (AP-32, *n* = 5), RM-treated PD (RM, *n* = 5), and A-RM-treated PD (A-RM, *n* = 5). One-way ANOVA was performed after grouping to ensure the equal animal grouping. After the animal grouping, rats were given either AP-32 (1.03 × 10^9^ CFU/kgBW), RM (62 mg/kgBW), or A-RM (1.03 × 10^9^ CFU/kgBW of AP-32 mixed with 62 mg/kgBW of RM) once a day for 8 weeks by oral gavage administration. L-DOPA treatment (8 mg/kgBW of L-DOPA + 15 mg/kgBW benserazide dissolved in 0.9% NaCl) was performed by intraperitoneal (i.p.) injection 3 times/week (Sgroi et al., 2014)started from animal grouping until 8 consecutive weeks. Every day, the food and water intakes by rats were recorded, and their bodyweights (BW) were measured weekly. Apomorphine-induced rotation test was repeated at 4 and 8 weeks after supplementations. Catwalk-gait analysis and body composition were evaluated after 8 weeks of supplementations. Rats were sacrificed afterward. The timeline of the study is shown in Figure 1. All animal procedures were approved by the Taipei Medical University Animal Care and Use Committee of Panel (IACUC/IACUP) (approval no. LAC-2020-0183) and were conducted in accordance with the Taiwan code of practice for the care and use of animals for scientific purposes.

### Unilateral 6-OHDA Injection Into the Right MFB

Induction of PD using unilateral 6-OHDA injection into the right MFB was modified from Hoffer et al. (1994). Rats were anesthetized through i.p. injection of tiletamine with zolazepam (20–40 mg/kgBW) (Zoletil^®^, Virbac, New Zealand) and xylazine (5–10 mg/kgBW) (Rompun^®^, Bayer HealthCare, Germany). Then, rats were fixed in a stereotaxic apparatus (David Kopf Instruments, Tujunga, CA, United States) by positioning their forehead flat. After exposing the skull by surgical incision, 1-mm burr hole was drilled into the right medial forebrain bundle (MFB) with the coordinates: anteroposterior (AP) −4.4 mm from the bregma; mediolateral (ML) 1.2 mm from the midline; and dorsoventral (DV) −7.8 mm. Subsequently, 6−OHDA (9 μg per rat in 3 μl of 0.9% NaCl + 0.02% (w/v) ascorbic acid) (Sigma-Aldrich, Darmstadt, Germany) was infused by an infusion pump through a 10 μl Hamilton syringe at 0.2 μl/min, constant flow rate. The implanted syringe was left still for an additional 5 min after infusion and then retracted slowly. Sham−operated rats underwent the same procedure with 3 μl of the vehicle (0.9% NaCl + 0.02% (w/v) ascorbic acid). To reduce variability caused by 6-OHDA degradation, the 6-OHDA solution was freshly prepared in ascorbic acid solution, kept at 4°C, and protected from light (Sanchez et al., 2009). The fresh solution was used within 1 h. During the surgery and recovery procedures, animals were maintained warm using a heating pad.

### Serum Biochemical Analysis

Blood was immediately collected by cardiac puncture, and the rats were immediately sacrificed after 8-weeks of supplementation. Blood was centrifuged at 3,500 x *g* for 10 min at 4°C to obtain the serum. Serum total cholesterol (TCHO), triglycerides (TG), high-density lipoprotein cholesterol (HDL), aspartate aminotransferase (AST), alanine aminotransferase (ALT), albumin (ALB), and total protein (TP) were measured using an autoanalyzer (Fujifilm NX500i, Tokyo, Japan).

### Evaluation of Neurodegeneration Process and Motor Function

#### Apomorphine-Induced Rotation Test

Apomorphine-induced rotation test was performed at 6 weeks after unilateral 6-OHDA lesions to assess the variability of the unilateral 6-OHDA-induced PD rats. Rats were assigned separately into a square cage. A dopamine receptor agonist, apomorphine (0.5 mg/kgBW in 0.9% NaCl and 1% ascorbic acid) (Sigma-Aldrich, Darmstadt, Germany), was injected subcutaneously to trigger contralateral rotation. Contralateral rotations were recorded for 1 hour. Contralateral rotations induced by apomorphine reflect the dopaminergic neuron loss. The test was repeated at 4 and 8 weeks after supplementations to assess the supplementations effects.

#### Immunohistochemistry Staining and Image Analysis

Immunohistochemistry staining was used to evaluate tyrosine hydroxylase (TH) presence in the striatum and SNc. TH is the specific marker to determine dopaminergic neuron number. Briefly, each brain was cut into coronal slices containing striatum (interaural 9.20 mm and bregma 0.20 mm) and SNc (interaural 3.40 mm and bregma −5.60 mm), then incubated in 4% formaldehyde. The free-floating sections were incubated in a 0.3% hydrogen peroxide solution for 10 min under light-protected condition light, to block internal peroxidases. After that, sections were incubated in PBS containing 0.5% Triton X-100, 4% BSA at room temperature for one hour. Sections were then left to incubate with rabbit polyclonal anti-TH (1:300) (Elabscience, US, Cat. No. E-AB-70077) in PBS containing 0.4% Triton X-100 at 4°C overnight. The primary antibody was omitted in control sections. After incubation for two hours at room temperature with goat anti-rabbit IgGHRP (Sigma, B7401), 1:100 in PBS containing 0.4% Triton X100, immunocomplexes were revealed using 3,3′-diamino-benzidine (DAB Substrate Kit for Peroxidase, Vector) as the chromogen. After washing extensively, the sections were dehydrated and mounted on slides using Permount (Fisher Scientific, United States) mounting media. Slides imaging was performed using pathology slide scanner Motic EasyScan hardware and software (Motic, Hong Kong). The intensity of TH + level was then quantified using ImageJ. The intensity of TH + level of lesioned side was standardized with the respected non-lesioned side (contralateral side).

#### Catwalk Quantitative Gait Analysis Test

A Catwalk-gait test was performed to evaluate the gait performance after 8 weeks of supplementations. The Catwalk-gait test was done on a quantitative gait analysis system, GaitLab ViewPoint (ViewPoint, China) hardware and software. The apparatus consisted of a raised glass floor with a green light. On the bottom floor of the walkway, a high-speed video camera is placed to record the green light reflected from each point of contact with the animal at 100 frames per second. The pattern of green light was interpreted by custom software and translated into a quantitative assessment of the rat’s paw print. The following variables were obtained from Catwalk-gait analysis: speed (speed started from entering until leaving the walkway, cm/s), stride length (distance between the same paw, cm), stride frequency (number of strides per second, strides/sec), stance time (the time the animal lays a paw on floor, secs), duty factor (ratio of a paw frequency and total time), asymmetry gait analysis (left pair lag, secs; right pair lag, secs; left pair gap, cm; right pair gap, cm), and intensity sum.

### Evaluation of Mitochondrial Function and Energy Metabolism

#### Mitochondrial Function by Seahorse Bioscience XFe24 Extracellular Flux Analysis

Mitochondrial function was evaluated by measuring real time basal oxygen consumption rates (OCR) and extracellular acidification rate (ECAR) with Seahorse Bioscience XFe24 extracellular flux analyzer. OCR measures mitochondrial respiration efficacy and ECAR measures glycolysis efficacy. After sacrifice, fresh brains and left soleus muscles were transferred into an XFe24 islet capture microplate (Seahorse Bioscience, United States, Cat. No. 101122-100). The Seahorse XFe24 performs repeated measurement of oxygen and proton concentrations surrounding tissues in the assay medium using oxygen and hydrogen ion-sensitive fluorophores. Briefly, inside a non-carbon dioxide incubator the assay cartridge plate (Seahorse Bioscience, United States, Cat. No. 100867-100) was hydrated using an XF calibration solution (Seahorse Bioscience, United States, Cat. No. 100867-000) for overnight at 37°C. For the experiments, Seahorse XF Assay medium (Seahorse Bioscience, United States, Cat. No. 102365-100), containing 5.0 mmol/l glucose and 2.0 mmol/l pyruvate at pH 7.4 and 37°C, was used to minimize any movement during the assay, isolated tissues were placed at the bottom of the islet plates and covered with a screen. 525 μl of XF assay medium was then added into the wells and maintained at 37°C in a non-carbon dioxide incubator for 20 min. After the incubation, the islet plate was placed inside the analyzer instrument. Three cycles were performed to obtain basal mitochondria respiration and glycolysis rate. After the Seahorse experiment, protein concentration in each well was then determined. The tissues from all islet plate wells were transferred into individual 1.5 ml microtubes containing 5 μl/ml (each) of NP40 lysis buffer (Invitrogen, Frederick, MD), proteinase, and phosphatase inhibitor at 4°C (Sigma Aldrich, St. Louis, MO). Tissues were homogenized and then centrifuged for 10 min at 1,000 x *g*. The supernatant was separated and then used for the Bradford protein quantification assay (Thermo Scientific, Rockford, IL). Sample duplicates and bovine serum albumin (BSA) standards (Sigma-Aldrich, Darmstadt, Germany) were load into a 96-well plate with the following concentrations (mg/ml): 0, 25, 125, 250, 500, 750, 1,000, 1,500, and 2,000. The Bradford reagents (Sigma-Aldrich, Darmstadt, Germany) were added to the wells containing either samples or standards. Afterward, the plate was incubated for 5 min at room temperature, and 595 nm was used to read the absorbance of the samples and standards. Concentration of samples was finally calculated by using standard curve and interpolation technique.

#### Dual-Energy X-Ray Absorptiometry (DXA)

After 8 weeks of supplementation, the body composition of each rat was assessed to determine total body fat, muscle mass, and bone mineral density by using DXA (Lunar Hologic, GE Healthcare, Wisconsin, United States) hardware and software. To ensure rats were motionless during scanning, rats were anesthetized using zoletil (20-40 mg/kgBW) and xylazine (5–10 mg/kgBW) by i.p. injection. With their face facing downwards, the rats were placed on the scanning bed. Nose to tail scanning was conducted to obtain complete body composition measurement.

### Antioxidant Activity

Glutathione peroxidase (GPx) and superoxide dismutase (SOD) activities were measured in serum using colorimetric assay using GPx and SOD assay kits (BioVision, California, United States) according to the manufacturer’s instructions. The GPx and SOD activities were calculated using an equation obtained from linear regression of the standard curve.

### Fecal Microbial Metabolites Analysis by High-Performance Liquid Chromatography (HPLC)

Fresh fecal samples were collected during the eighth week. Determination of microbial metabolites consisted of six SCFAs (acetic, propionic, isobutyric, butyric, isovaleric, and valeric acids). SCFAs were determined by HPLC, following a previous study (Granado-Serrano et al., 2019). Briefly, SCFAs were extracted using 70% ethanol (5 mL of ethanol for 200 mg of the sample), then mixed and centrifuged at 20°C, 2500 rpm, 10 min. The supernatant was mixed with 2-ethylbutyric acid, pyridine, 1-EDC-HCl, and 2-NPH-HCl, then re-acted to 60°C for 20 min. The mixture was then mixed with potassium hydroxide, reacted at 60°C for 20 min, and cooled down. Next, the mixture was shaken with a phosphoric acid aqueous solution and ether for 3 min, then centrifuged to collect the ether layer. The ether layer was shaken with water for 3 min then centrifuged. Nitrogen gas was used to eliminate ether. Lastly, HPLC (column temperature 50°C, a flow rate of 1.1 mL/min, measurement wavelength 400 nm) was used after dissolving the obtained fatty acid hydrazide with methanol.

### Statistical Analyses

Statistical analyses were performed using SPSS Statistics 19 software. Differences were evaluated using one-way ANOVA followed by Tukey HSD *post hoc* test. Results are expressed as mean ± standard error of the mean (SEM). Significance was determined as p < 0.05.

## Results

### Serum Biochemical Profiles After 8 Weeks of AP-32, RM, and A-RM Supplementation

Levels of TCHO, TG, HDL, AST, ALT, ALB, and TP were measured in serum after 8-weeks of treatment. There was no significant difference among groups in serum bio-chemical profiles (Table 1), suggesting that AP-32, RM, and A-RM will not affect the serum biochemical value.

**TABLE 1:** The effects of AP-32, RM, and A-RM on serum biochemical profiles after 8 weeks of treatment in 6-OHDA-induced PD rats.

**Biochemistry levels**	**NC**	**PD**	**L**	**AP-32**	**RM**	**A-RM**
TCHO (mg/dL)	81.8 ± 21.2	84.4 ± 14.7	75.8 ± 13.6	85 ± 17.5	71.7 ± 11.1	77.3 ± 11.1
TG (mg/dL)	108.8 ± 46.7	94 ± 44.7	96.8 ± 46.2	104.7 ± 25.6	107.7 ± 14.9	95.0 ± 56.4
HDL (mg/dL)	44.8 ± 9.6	46.7 ± 15.0	46.8 ± 6.3	39.2 ± 6.0	39.7 ± 11.1	33.3 ± 3.8
AST (u/L)	183.3 ± 54.5	247 ± 55.3	185 ± 52.0	141.3.8 ± 31.1	274.3 ± 50.5	318.0 ± 50.8
ALT (u/L)	47.8 ± 4.1	54.3 ± 8.0	43.5 ± 5.2	43 ± 3.7	46.7 ± 5.3	59.3 ± 9.9
ALB (g/dL)	4.6 ± 0.9	3.7 ± 0.2	4.3 ± 0.7	3.9 ± 0.4	3.5 ± 0.1	3.8 ± 0.2
TP (g/dL)	7.2 ± 0.8	6.4 ± 0.3	6.8 ± 0.6	6.4 ± 0.3	6.1 ± 0.2	6.4 ± 0.2

*All data are expressed mean ± SEM (*n* = 5 rats/group). There is no significant difference between groups. NC, normal control; PD, untreated PD as negative control; L, PD treated with 8 mg of L-DOPA as a positive control; AP-32, PD treated with 1.03 × 109 CFU/kg BW of AP-32; RM, PD treated with 62 mg/kgBW of residues of microbial culture medium RM; A-RM, PD treated with AP-32 and 62 mg/kgBW of residues of microbial culture medium RM. TCHO, Total Cholesterol; TG, Triglycerides; HDL, High Density Lipoprotein-Cholesterol; AST, Aspartate Aminotransferase; ALT, Alanine Aminotransferase; ALB, Albumin; TP, Total Protein.*

### Supplementation of Probiotic *L. salivarius* AP-32, Prebiotic RM, and Combination A-RM Attenuated Neurodegenerative Process

### Apomorphine-Induced Rotation

Contralateral rotation induced by apomorphine could reflect the dopaminergic neuron loss after unilateral 6-OHDA injection into MFB. Six weeks after the injection, rats were exhibited more than 200 contralateral rotations/hour (Table 2). After 4 weeks of supplementations, contralateral rotations were decreased by 31.6 ± 7.3% in AP-32, 29.1 ± 12% in RM, and 27.9 ± 6.3% in A-RM-treated group. The contralateral rotations continuously decreased after 8 weeks of supplementations, with reduction of 66.7 ± 10.7%, 51.3 ± 8.8%, and 60.1 ± 8.3% in AP-32, RM, and A-RM, respectively. Compared to untreated PD group, supplementation of AP-32, RM, and A-RM significantly decreased contralateral rotations after 4- and 8-weeks supplementation. There is no significant difference between untreated PD and L-DOPA-treated group.

**TABLE 2:** AP-32, RM, and A-RM decreased contralateral rotation in apomorphine-induced rotation test after 4 and 8 weeks.

**Week**	**NC**	**PD**	**L**	**AP-32**	**RM**	**A-RM**
6^th^	0.0 ± 0.0	221.0 ± 13.9^#^	220.4 ± 9.3	223.8 ± 16.1	221.0 ± 12.1	214.8 ± 12.4
10^th^	0.0 ± 0.0	258.2 ± 31.3^#^	233.6 ± 20.6	187.6 ± 24.8*	191.4 ± 27.6*	194.0 ± 20.8*
14^th^	0.0 ± 0.0	269.6 ± 29.5^#^	258.4 ± 23.3	90.2 ± 24.6*	131.4 ± 20.8*	107.0 ± 21.2*

Apomorphine-induced rotation test was conducted at 6, 10, and 14 weeks after 6-OHDA lesion. Data are expressed mean ± SEM (*n* = 5 rats/group). ^#^*p* < 0.05 PD group compared to NC group. *p < 0.05 L, AP-32, RM, and A-RM groups compared to PD group. There is no significant difference between AP-32, RM, and A-RM. One-way ANOVA with Tukey’s *post hoc* test. BW, Bodyweight; FCE, Food Consumption Efficiency (an increase of BW/total calorie intake).

#### Neuroprotective Effect

**FIGURE 2:**
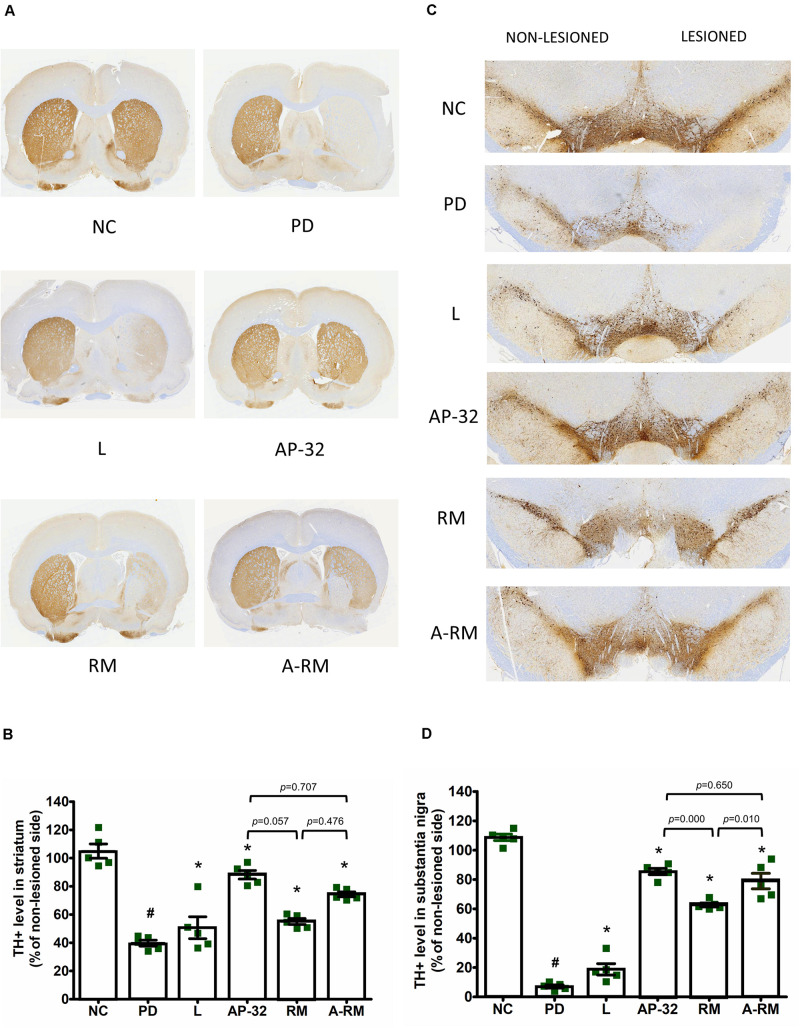
Supplementation of AP-32, RM, and A-RM rescued the 6-OHDA-mediated reduction of dopaminergic neurons in the striatum and substantia nigra pars compacta (SNc) after 8-weeks of supplementations. **(A)** A representative of the immunohistochemical staining for TH (dopaminergic neuron marker) in the striatum. **(B)** Quantitative analysis of the number of TH-positive cells in the striatum. **(C)** A representative of the immunohistochemical staining for TH in SNc. **(D)** Quantitative analysis of the number of TH-positive cells in the SNc. The intensity of the lesioned side was standardized with the non-lesioned side and reported as mean ± SEM (*n* = 5 rats/group). #*p* < 0.05 PD group compared to NC group. *p < 0.05 L, AP-32, RM, and A-RM groups compared to PD group. Data were evaluated by one-way ANOVA with Tukey’s *post hoc* test.

Dopaminergic neurons were evaluated using a specific marker, TH. TH + level of the untreated PD group showed dramatic reductions in striatum (Figures 2A,B) and SNc (Figures 2C,D) of the 6-OHDA-lesioned side. Supplementation of AP-32 and its combination with residual medium (A-RM) prevented the loss of TH + intensity level in the striatum and SNc induced by unilateral 6-OHDA injection, indicating neuroprotective effects performed by these supplementations. A significant increase in the TH + level of the RM group was only observed in the SNc but not in the striatum. Supplementation of AP-32 for 8 weeks showed slightly higher TH + level in the striatum and SNc than RM. Here we also noted that RM seemed to have a suppression effect on AP-32, as seen in a slight decrease of TH + level in a group supplemented with A-RM.

### Evaluation of Gait Function

**FIGURE 3:**
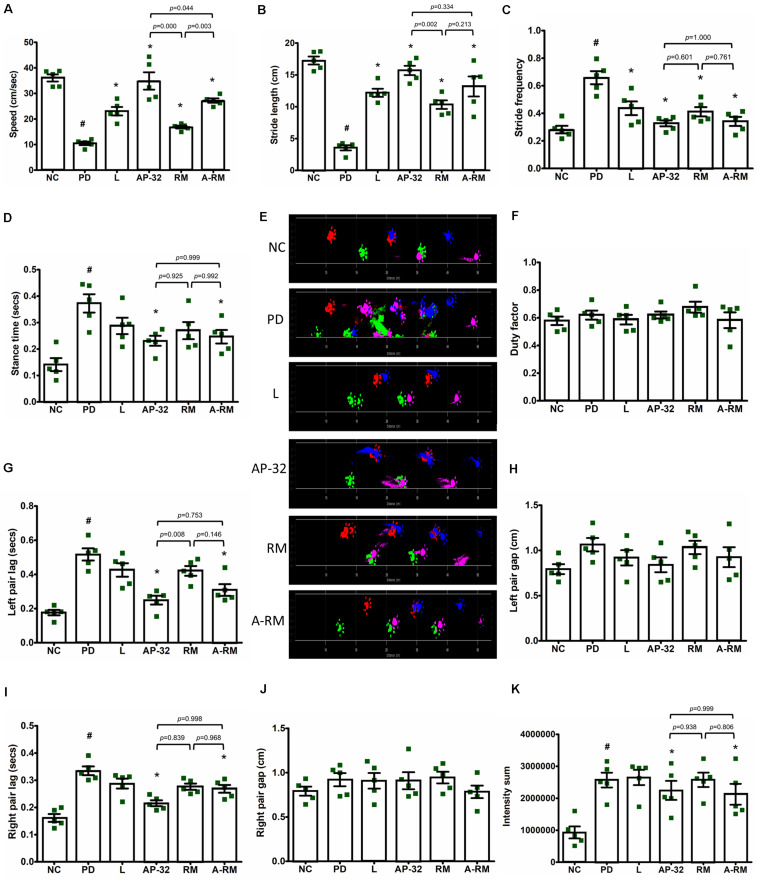
Dynamic parameters for gait analysis in 6-OHDA-induced PD rats. **(A)** Speed started from entering until leaving the walkway. **(B)** Stride length. **(C)** Stride frequency. **(D)** Stance time. **(E)** Section from original footprints recorded after 8-weeks supplementations; pink, front left; blue, front right; green, hind left; red, hind right. **(F)** Duty factor. **(G)** Left pair lag. **(H)** Left pair gap. **(I)** Right pair lag. **(J)** Right pair gap. **(K)** Intensity sum. All data are expressed as mean ± SEM (*n* = 5 rats/group). ^#^*p* < 0.05 PD group compared to NC group. *p < 0.05 L, AP-32, RM, and A-RM groups compared to PD group. Data were evaluated by one-way ANOVA with Tukey’s *post hoc* test.

Unilateral 6-OHDA MFB injection induced gait dysfunction as seen in dynamic parameters in Catwalk-gait analysis (Figure 3). Significant decreases of speed (Figure 3A) and stride length (Figure 3B) were found in untreated PD group. However, supplementation of probiotic AP-32, RM, and A-RM for 8 weeks could significantly increase the locomotor speed and stride length of PD rats. AP-32 supplementation shows better speed performance than RM and A-RM. Stride frequency (Figure 3C) was high in untreated PD group and decreased in AP-32, RM, A-RM, and L-DOPA-treated group. Stance time (Figure 3D) was increased by unilateral 6-OHDA injection but significantly reduced by AP-32 and A-RM supplementation. Altogether, the gait analysis results suggest that unilateral 6-OHDA injection into MFB impaired the gait function by decreasing the ability to move faster and further. But supplementation of AP-32, RM, and A-RM showed improvement of the gait function. Furthermore, supplementation of AP-32 and A-RM showed reduced left pair lag compared to the untreated PD group (Figure 3G). This result is corresponded with the preserved TH + level in AP-32 and A-RM-treated group, suggesting that unilateral 6-OHDA injection induced asymmetry gait dysfunction and both supplementations could alter the dysfunction.

### Supplementation of Probiotic *L. salivarius* AP-32, Prebiotic RM, and Combination A-RM Restored Mitochondrial Function and Energy Metabolism

#### Mitochondrial Function Analysis

**FIGURE 4:**
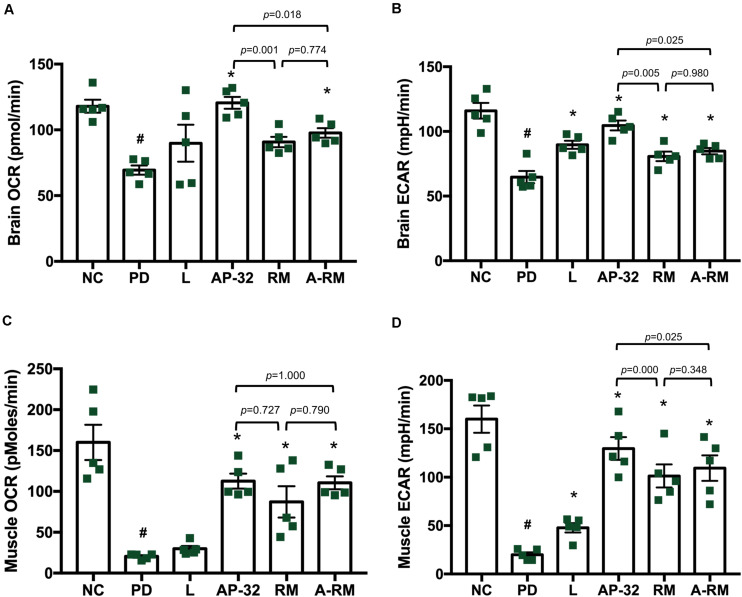
The effect of AP-32, RM, and A-RM on mitochondrial function in the brain and soleus muscle after 8-weeks of supplementation. Mitochondrial function was evaluated by basal mitochondria respiration, oxygen consumption rate (OCR), and basal glycolysis rate, and basal extracellular acidification flux (ECAR). **(A,B)** The mitochondrial activates in the brain, including striatum and SNc. **(C,D)** The mitochondrial actives in the soleus muscle. All data are expressed as mean ± SEM (*n* = 5 rats/group). #*p* < 0.05 PD group compared to NC group. *p < 0.05 L, AP-32, RM, and A-RM groups compared to PD group. Data were evaluated by one-way ANOVA with Tukey’s *post hoc* test.

Mitochondrial function was evaluated in the brain and soleus muscle through measuring OCR and ECAR. In the brain, including striatum and SNc, mitochondrial function was decreased in the untreated PD group, marked by decreases in OCR and ECAR (Figures 4A,B). However, supplementation of AP-32 and A-RM in-creased OCR and ECAR. Group treated with L-DOPA and RM only showed an in-crease in ECAR and not in OCR. Supplementation of AP-32 significantly showed the highest OCR and ECAR in the brain compared to RM and A-RM, indicating that AP-32 could improve mitochondrial function in the brain better than RM and A-RM. This is consistent with TH immunohistochemistry results that show AP-32 exerted the best neuroprotective effect among all supplementation groups. Subsequently, the untreated PD group shows a decrease in mitochondrial function in the soleus muscles as evaluated by OCR and ECAR values (Figures 4C,D). Supplementation of AP-32, RM, and A-RM could reverse the decreases induced by 6-OHDA, with AP-32 showed the best results. The OCR and ECAR results in the brain and soleus muscle further supported an evidence that AP-32 exerted better effects than RM and A-RM.

#### BW Gain, Food Consumption Efficiency (FCE), Water Intake, and Body Composition

The BW of the rats was observed after 6-OHDA injection. Each group gained BW during the experiment period. However, the untreated PD group showed less BW gain than other groups (Table 3). Probiotic *L. salivarius* AP-32 and combination A-RM treatment group showed significantly higher BW gain than the untreated PD group. The supplementation of AP-32 for 8 weeks also resulted in greater BW gain than RM and A-RM. It indicated that AP-32 could prevent BW loss better than RM and A-RM.

**TABLE 3:** The effect of AP-32, RM, and A-RM on BW gain, food intake, food conversion efficiency, and water intake in 6-OHDA-induced PD rats.

**Parameter**	**NC**	**PD**	**L**	**AP-32**	**RM**	**A-RM**
BW gain (g)/rat	320.2 ± 9.5	127.6 ± 14.2^#^	200.8 ± 14.7*	295.0 ± 25.4*	227.6 ± 8.5*^[*dollar*]^	250.4 ± 14.7*^[*dollar*]^
Food intake (Kcal)/day/rat	112.8 ± 14.4	101.4 ± 9.2	107.1 ± 9.3	105.0 ± 7.7	103.5 ± 8.4	102.8 ± 6.9
FCE (%)	2.9 ± 0.3	1.3 ± 0.2^#^	1.9 ± 0.2*	2.8 ± 0.3*	2.2 ± 0.2*^[*dollar*]^	2.4 ± 0.1*
Water intake (ml)/day/rat	34.2 ± 2.7	33.9 ± 1.4	34.2 ± 1.7	34.9 ± 2.1	32.3 ± 3.2	34.3 ± 2.6

Data are expressed mean ± SEM (*n* = 5 rats/group). ^#^*p* < 0.05 PD group compared to NC group. *p < 0.05 L, AP-32, RM, and A-RM groups compared to PD group. ^$^*p* < 0.05 RM and A-RM groups compared to AP-32 group. One-way ANOVA with Tukey’s *post hoc* test. BW, Bodyweight; FCE, Food Consumption Efficiency (an increase of BW/total calorie intake).

There is no significant difference in water and food intake between groups (Table 3). The untreated PD group shows the lowest FCE compared to other groups. These results indicated that probiotic AP-32, prebiotic RM, and combination A-RM could increase the efficiency of food consumption and attenuate BW loss induced by 6-OHDA.

**FIGURE 5:**
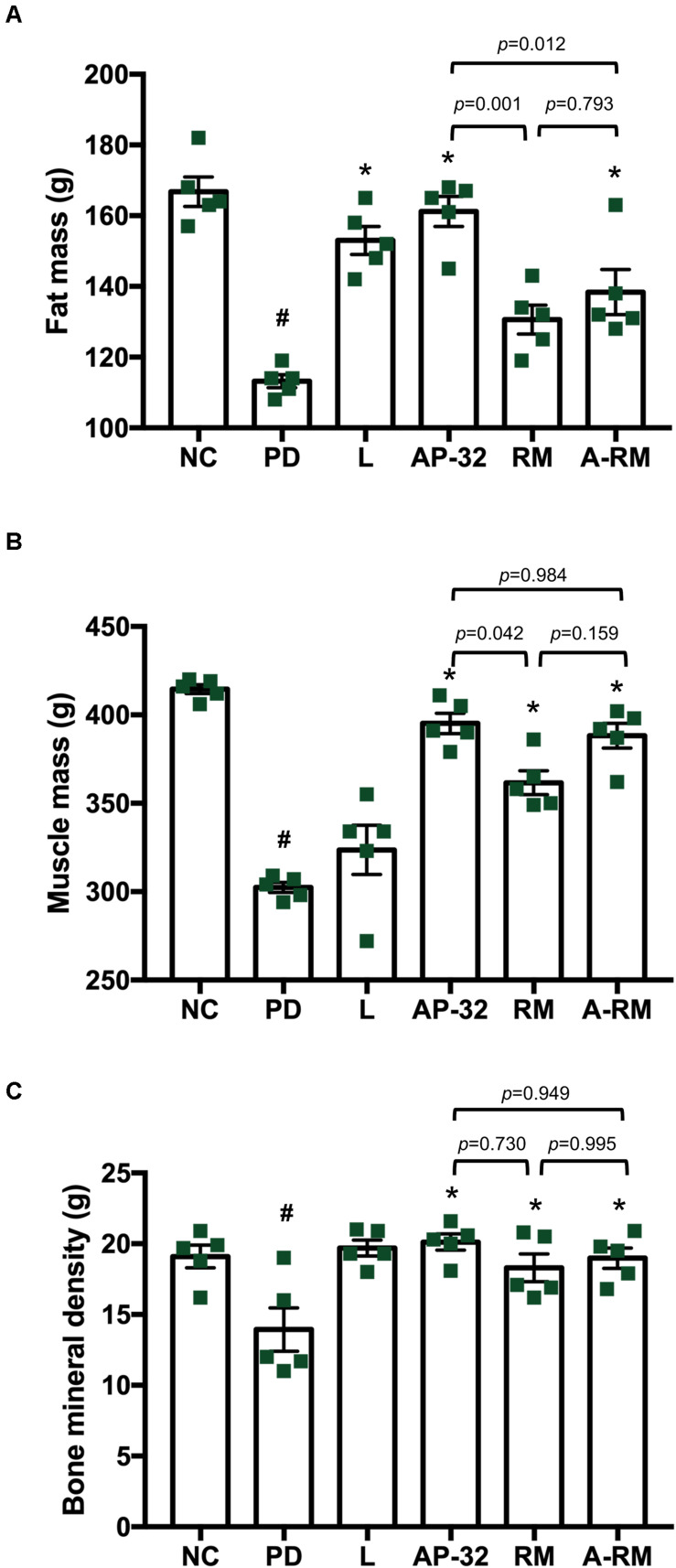
Body composition of rats after 8 weeks of treatments. Body composition was evaluated by Dual-Energy X-Ray Absorptiometry (DXA), which includes **(A)** fat mass, **(B)** muscle mass, and **(C)** bone mineral density. All data are expressed as mean ± SEM (*n* = 5 rats/group). *p < 0.05 compared to PD group. Data were evaluated by one-way ANOVA with Tukey’s *post hoc* test. ^#^*p* < 0.05 PD group compared to NC group.

Consistent with BW declines, body composition measured through DXA (Figure 5) showed that the untreated PD group had lower total fat mass, muscle mass, and bone mineral density than the healthy group. Supplementation of probiotic AP-32 and RM prevented the decreases, with AP-32 performed the best among all treatments, followed by A-RM and RM. Muscle mass was preserved in groups treated with AP-32, RM, and RM, indicating their ability to prevent muscle atrophy in 6-OHDA-induced PD rats. Together, these results suggest that AP-32, RM, and A-RM could prevent BW loss, increase FCE, and maintain body composition, with AP-32 showed the best result among supplementation groups. Here we also observed significant increases of fat mass and bone mineral density mass, but not muscle mass in L-DOPA-treated group.

### Supplementation of Probiotic *L. salivarius* AP-32 Enhanced Antioxidative Enzyme Activities

**FIGURE 6:**
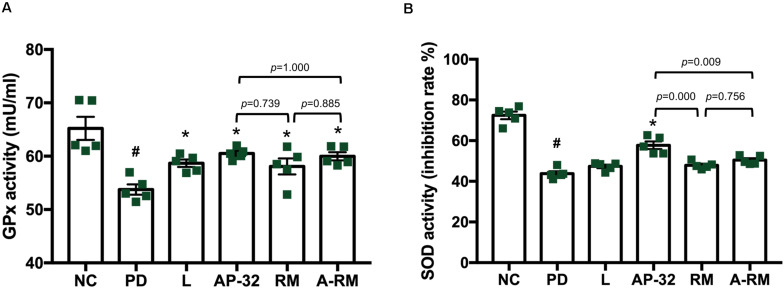
The effect of AP-32, RM, and ARM on antioxidative enzyme activities in serum. **(A)** Glutathione peroxidase (GPx) activity, **(B)** superoxide dismutase (SOD) activity. Data are expressed as mean ± SEM (*n* = 5 rats/group). #*p* < 0.05 PD group compared to NC group. *p < 0.05 L, AP-32, RM, and A-RM groups compared to PD group. All data were evaluated by one-way ANOVA with Tukey’s *post hoc* test.

Serum antioxidative enzyme activities of GPx and SOD were decreased in 6-OHDA-induced PD groups (Figure 6). Treatment of AP-32, RM, A-RM, and L-DOPA significantly increased GPx activity in serum. However, only the PD group treated with AP-32 showed significant improvement in SOD activity. The combination of AP-32 and RM could not exert the similar effects exerted by supplementation of AP-32 alone. It suggests that AP-32 was the better supplement in increasing antioxidative enzyme activities compared to RM and A-RM. The results are also consistent with results of TH immunohistochemistry and gait function evaluation which indicate that AP-32 performed the best among all treatments.

### The Fecal SCFAs Were Increased by Supplementation of Probiotic *L. salivarius* AP-32, Prebiotic RM, and Combination A-RM

Induction of PD with 6-OHDA neurotoxin reduced microbial metabolites, SCFAs, in fecal samples (Table 4). Supplementation of AP-32, RM, and their combination (A-RM) elevated SCFAs. AP-32, RM, and A-RM supplementations showed significantly higher total acid, propionic acid, and butyric acid than the untreated PD group. Isovaleric acid only increased by supplementation of AP-32 and RM. Meanwhile, isobutyric acid only elevated in the group supplemented with AP-32. Among all supplementation group, AP-32 showed slightly higher isobutyric acid, butyric acid, and valeric acid.

**TABLE 4:** The effect of AP-32, RM, and A-RM on fecal SCFAs profiles after 8 weeks of treatments.

**SCFAs (mM)**	**NC**	**PD**	**L**	**AP-32**	**RM**	**A-RM**
Total acid	6.43 ± 0.64	4.48 ± 1.37^#^	5.38 ± 1.28	6.39 ± 0.42*	6.42 ± 0.87*	6.50 ± 1.73*
Acetic acid	4.33 ± 0.61	3.63 ± 1.36	3.99 ± 0.92	4.49 ± 0.53	4.96 ± 0.79	4.92 ± 0.90
Propionic acid	1.06 ± 0.06	0.58 ± 0.16^#^	0.95 ± 0.12*	0.93 ± 0.10*	1.00 ± 0.07*	1.16 ± 0.37*
Isobutyric acid	0.03 ± 0.01	0.03 ± 0.00	0.03 ± 0.01	0.05 ± 0.01*	0.04 ± 0.02	0.04 ± 0.01
Butyric acid	1.80 ± 0.13	0.54 ± 0.11^#^	0.90 ± 0.38	1.57 ± 0.38*	1.23 ± 0.21*	1.21 ± 0.61*
Isovaleric acid	0.02 ± 0.00	0.01 ± 0.00	0.01 ± 0.01	0.03 ± 0.01*	0.03 ± 0.01*	0.02 ± 0.00
Valeric acid	0.08 ± 0.01	0.06 ± 0.01	0.05 ± 0.01	0.07 ± 0.02	0.05 ± 0.03	0.05 ± 0.02

Data are expressed as mean ± SEM (*n* = 5 rats/group). ^#^*p* < 0.05 PD group compared to NC group. *p < 0.05 L, AP-32, RM, and A-RM groups compared to PD group. There is no significant difference between AP-32, RM, and A-RM. One-way ANOVA with Tukey’s *post hoc* test. SCFAs, short-chain fatty acids.

## Discussion

**FIGURE 7:**
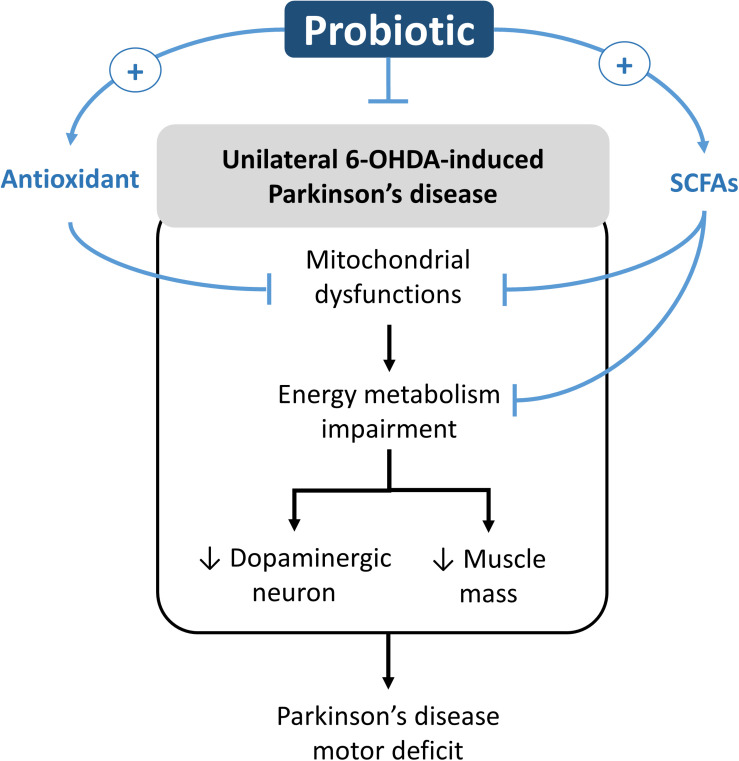
Potential neurorestoration molecular pathway of probiotic in facilitating recovery of motor deficit in unilateral 6-OHDA-induced Parkinson’s disease.

In this study, we found that the 8-week supplementation of probiotic [*Lactobacillus salivarius* subsp. *salicinius* AP-32 (AP-32)], prebiotic [residual medium (RM)], and the symbiotic [combination of AP-32 and RM (A-RM)] significantly prevented dopaminergic neuron loss along with improved motor function in PD rats. Along with their neuroprotective effects, the supplementations induced marked increase in the following: mitochondrial activities and glycolysis in the brain and muscle, antioxidative enzymes activities in the serum, and altered SCFAs profile in fecal samples. We noted that the supplementations also resulted in restoration of muscle mass and accompanied improvement of motor function. Overall, the supplementations were found to prevent the 6-OHDA-induced progression of PD and its associated symptoms, where AP-32 supplementation was found to yield the best results. The probiotic supplementation could play as an active modulator in regulating the onset of motor deficit progression in unilateral 6-OHDA-induced PD rats. The possible neurorestoration molecular pathway of probiotic is shown in Figure 7.

From our understanding, there could be two possible pathways underlying the neuroprotective effects of probiotic AP-32, prebiotic RM, and symbiotic A-RM. First, the increases of mitochondrial activities and glycolysis (Figure 4) by supplementation of probiotic AP-32, prebiotic RM, and symbiotic A-RM might increase energy metabolism in the brain and muscle, thus prevented the loss of dopaminergic neurons (Figure 2) and muscle atrophy (Figure 5). Second, the modulation of the SCFAs production (Table 4) and increase in antioxidative enzyme activities (Figure 6) induced by these supplementations might have played a vital role in protecting the mitochondria against reactive oxygen species (ROS).

Neurodegenerative diseases, like PD, have been associated with impaired energy metabolism in the brain, which results in excessive neuronal deaths. Mitochondrial respiration and glycolysis are the two important metabolic pathways during energy metabolism in neurons. In a PD brain, mitochondrial electron transport chain reaction in tricarboxylic acid (TCA) cycle is known to malfunction, which results in decreased mitochondrial respiration (Schapira et al., 1990). Also, the reduction in glycolysis process is associated with many of neurodegenerative diseases. Dysfunctions in mitochondrial respiration and glycolysis reduction lead to declined ATP production (Tait and Green, 2013). As neurons are high energy demanding cells (Verstreken et al., 2005), the deficit in energy production and high energy demand can lead to energy starvation in the neurons, impairing their function and eventual death via apoptotic pathway (Tait and Green, 2013). In this study, we found that there were increases in mitochondrial respiration activities and glycolysis in the brain and muscle of rats supplemented with probiotic AP-32, prebiotic RM, and symbiotic A-RM (Figure 4). Thus, our findings indicate that these supplements might facilitate the modulation of energy metabolism for meeting the energy demand in the brain and muscles, which might have prevented the dopaminergic neuron loss and muscle atrophy.

Mitochondrial dysfunctions can lead to increased oxidative stress from the generation of ROS, such as superoxide anion and hydrogen peroxide. This can further increase neuronal deaths, a characteristic typically found in neurodegenerative diseases (Shukla et al., 2011; Federico et al., 2012; Subramaniam and Chesselet, 2013). In normal conditions, antioxidants such as SOD and GPx are innately produced to neutralize the oxidative stress. However, in the pathological state of PD, neurons fail to produce sufficient levels of antioxidants to counterbalance the ROS (Di Pietro et al., 2014; Cho et al., 2016; Feuerstein et al., 2016). The cumulative increase of oxidative stress damages the mitochondria and interferes with the energy production process (Shukla et al., 2011). Therefore, restoring the balance between ROS and antioxidants can be crucial for preventing the damage in dopaminergic neurons of PD (Shukla et al., 2011; Poewe et al., 2017). In our study, we observed that 6-OHDA decreased oxidative stress-related antioxidant enzymes, SOD and GPx, in the serum (Figure 6). However, long-term supplementation of probiotic AP-32 resulted in increase of SOD and GPx activity levels in the serum of 6-OHDA treated rats. The increase of serum antioxidant enzymes indicates that these supplementations could indirectly have a protective effect against ROS to decrease oxidative stress in the brain (Cho et al., 2016). It was observed that prebiotic RM and symbiotic A-RM could only significantly elevate GPx level. The results thus indicate that supplementing pure AP-32 can generate a better antioxidant production capacity *in vivo* than only supplementing the prebiotic metabolites (RM) extracted from its culture.

Upon analyzing the fecal samples obtained from the rats, it was determined that the probiotic AP-32, prebiotic RM, and symbiotic A-RM supplementation altered the SCFAs levels in the rodent’s gut (Table 4). SCFAs (e.g., acetate, propionate, and butyrate) generated from fermentation by gut microbiota in the large intestine are known to exert antioxidant properties, immunomodulatory function, and regulate energy metabolism (Koh et al., 2016; Schonfeld and Wojtczak, 2016; LeBlanc et al., 2017). The observed increases of butyric acid and propionic acid are likely to be involved in the elevation of antioxidative enzymes (SOD and GPx) in the group treated with AP-32 (and increase of GPx in RM and A-RM group). This suggests that AP-32 might reduce oxidative stress by increasing host antioxidant enzyme activities. Probiotics can decrease oxidative stress by producing anti-oxidative enzymes, stimulating antioxidant metabolites (e.g., folate, glutathione), up-regulate host anti-oxidative enzymes activities, trigger other signaling pathways (e.g., NFkB, PKC, MAPK, Nrf2), and down-regulate ROS-producing enzymes activities (Wang et al., 2017). Also, the antioxidant capacity induced by SCFAs might be involved in counteracting the mitochondria-destroying oxidative stress prevailing in a PD brain. With reduced oxidative stress, mitochondria could function properly to supply energy in the brain and muscles, supported by the finding of increased OCR and ECAR in the PD group treated with AP-32, RM, and A-RM (Figures 4A,B). In addition, SCFAs could up-regulate energy metabolism in the brain and muscle by serving as energy substrates (Lei et al., 2016; Schonfeld and Wojtczak, 2016; LeBlanc et al., 2017). Specifically, butyric acid is known to enhance mitochondrial activity by increasing oxygen consumption and glucose uptake (Clark and Mach, 2017), which could facilitate enhanced energy utilization in the brain. Thus, the prevention of dopaminergic neurons loss in striatum and SNc along with reduced of motor dysfunction could be a synergistic effect originating from the enhanced anti-oxidative enzymes, improved mitochondria activities, and up-regulated energy utilization in the brain and muscles.

We also noted that AP-32, RM, and A-RM supplementation prevented the 6-OHDA induced loss of bodyweight (Table 3), muscle atrophy, and bone density deprivation (Figure 5) in rats. The reduced muscle atrophy in the soleus muscle was also accompanied with improved gait function evaluated by the Catwalk-gait test (Figure 3). Supplementation of AP-32, RM, and A-RM increased mitochondrial function in soleus muscle (Figures 4C,D). Several studies have described that PD patients also suffer from loss of bodyweight, muscle atrophy and concomitant impairments in motor functions (Garber and Friedman, 2003; Falvo et al., 2008; Stevens-Lapsley et al., 2012). There is also a known association between PD progression and decreased bone density (Handa et al., 2019). Consistent with these findings, our 6-OHDA treated rats reproduced the marked decreases in muscle mass and bodyweight (Kim and Choe, 2010; Choe et al., 2011, 2012; Minalyan et al., 2019) as well as bone density (Ali et al., 2019; Handa et al., 2019) in neurotoxin-induced PD animal model. Supplementation of the probiotic AP-32 prevented muscle atrophy, weight loss, and bone density deprivation, without affecting the food and water consumption behavior in the rats (Table 3). These effects by AP-32 and RM might rely on their capacity to modulate gut microbiota composition and microbiota metabolites. Supplementation of AP-32 might favor the commensal bacteria inside the intestine and their metabolites, which influence energy production, redox balance, immune response, and mitochondrial biogenesis (Clark and Mach, 2017). Increases in SCFAs production could up-regulate energy metabolism in the muscle by serving as energy substrates (Newman and Verdin, 2014; Kesl et al., 2016; Lei et al., 2016). Butyric acid enhances mitochondrial activity by increasing oxygen consumption and glucose uptake (Gao et al., 2009), which could increase energy supply in the muscle. The up-regulation of mitochondria and energy utilization indicates the ability of probiotic AP-32 and its RM in preventing body weight loss and muscle atrophy (Gao et al., 2009; Hock and Kralli, 2009). This restored muscle mass might also contribute to the improvement of motor function (Visser et al., 2005; Curcio et al., 2016). Regarding restored bone density in groups supplemented with AP-32, RM, and A-RM, it might also be another benefit of an increase in SCFAs production upon AP-32 and RM supplementation. SCFAs are known to protect bone density mass through downregulation osteoclastogenesis and bone resorption (Lucas et al., 2018). Additionally, SCFAs can increase the absorption of calcium, which is important in bone formation, through calcium and hydrogen exchange in the gut (Trinidad et al., 1996).

In this study, we found that supplementation of bacterial RM and A-RM could also reduce dopaminergic neuron loss, motor dysfunctions, and muscle atrophy, along with increased GPx and fecal SCFAs. The observed increases of propionic acid and butyric acid in PD groups supplemented with RM and A-RM could enhance antioxidant capacity (Koh et al., 2016; Schonfeld and Wojtczak, 2016), as seen in elevated serum GPx activity. As the RM contained SCFAs and various amino acids, including branched-chain amino acids (BCAAs) (Supplementary Table 1), the BCAAs can increase mitochondrial energy production rate and mitochondrial DNA abundance (Tatpati et al., 2010), which might explain the increase in mitochondrial activity. Thus, supplementation of RM and A-RM could also preserve dopaminergic neuron and muscle mass, possibly through enhancing GPx, SCFAs, and energy metabolism.

Although all supplementation groups showed significant neuroprotective effects, but from the Catwalk results, SOD activity, bodyweight and mitochondrial activities, AP-32 showed the best results compared to RM and A-RM groups. After comparing the protective effects of AP-32 and A-RM, we concluded that AP-32 has a better performance on Catwalk results (Figure 3), SOD activity (Figure 6), bodyweight (Table 3) and mitochondrial activities (Figure 4). Only the speed parameter was found to significantly decrease in RM and A-RM while the other parameters also followed the same trend but no significant difference. From those observations, it appears that RM may affect the function of AP-32. It needs more experiment to gain more deep understanding the interaction between AP-32 and RM.

Here we also observed effects of L-DOPA treatment on serum GPx activity ECAR in the brain and soleus muscle, fat mass, and bone mineral density. L-DOPA has been described to exert antioxidative effects (Han et al., 1996; Exner et al., 2003; Jodko-Piórecka and Litwinienko, 2015; Recky et al., 2021), including glutathione (Zhu et al., 2020). The upregulated GPx might affect the energy metabolism and bone mineral density. Further, previous study reported that L-DOPA administration could increase relative 2-deoxyglucose uptake in unilateral 6-OHDA injected substantia nigra pars reticulata rats (Trugman and Wooten, 1986). This result might explain our observation shown in Figures 4B,D because L-DOPA could increase relative 2-deoxyglucose uptake which might increase the glucose utilization in SNc. The result presented in this study is also supported by Adams et al. (2008) in which they reported levodopa/benserazide treatment increased lactate and pyruvate accumulation in human skeletal muscle, along with reduced lactate-to-pyruvate ratio, indicating increased aerobic glycolysis. Since L-DOPA can inhibit glycogen synthesis, then the decreased glycogen synthase could result in intracellular accumulation of glucose-6-phosphate (G6P) (Smith et al., 2004). As the cell could not store it as glycogen, it might lead to glucose utilization pathway, glycolysis (increased ECAR). In adipose tissue, L-DOPA is known to decrease dialysate glycerol, which suggest lipolysis inhibition (Adams et al., 2008). It might explain the preserved fat mass in PD group treated with L-DOPA.

We observed significantly higher bone mineral density in L-DOPA-treated group than the PD group. This result is contradictive with previous studies which suggest L-DOPA treatment induces bone loss by increasing homocysteine (Hcy), catechol O-methyltransferase (COMT)-stimulated L-DOPA metabolite (Yasui et al., 2003; Gjesdal et al., 2006). However, Hcy-induced bone loss can be caused by oxidative stress that triggers osteoclastic bone resorption (Koh et al., 2006). In our data, L-group also show higher GPX activity than the PD group. The increase of GPX activity might reduce the oxidative stress and promote the osteoclastic bone resorption. Additionally, study by Wang et al. demonstrate that the protective effect of glutathione against 6-OHDA-induced bone marrow stromal cell death (Wang et al., 2011). Furthermore, dopamine deprivation can lead to bone loss in PD as the system failed to prevent hyperprolactinemia (Ali et al., 2019; Handa et al., 2019). In *in vivo* study by Handa et al. (2019), higher dose of L-DOPA indeed increased Hcy, suggesting that L-DOPA might affect bone density in dose-dependent manner.

Further experiments are required to establish a more direct mechanism in the brain with respect to the AP-32, RM, and A-RM effects, including gut microbiota composition. Our ongoing study is now focused on the effects of probiotics on gut function and gut microbiota composition alterations. In summary, long-term supplementation of AP-32 was found to be the best treatment for protecting dopaminergic neurons and improve motor functions in 6-OHDA-induced PD rats. This supplementation enhanced SCFAs in the gut along with increased anti-oxidative enzymes in the systemic system, which probably was responsible for the reduction of oxidative stress in the brain. The elevated production of these fatty acids in the gut could be responsible for the increased mitochondrial activities in the brain and muscles, and directly involved in increased energy utilization. Recovery of these functions possibly underlie the decreased dopaminergic neuron loss and muscle loss prevention leading to improved motor functions in 6-OHDA-induced PD rats.

## Conclusion

The results obtained from our study reveal that long-term supplementation of probiotic *Lactobacillus salivarius* subsp. *salicinius* AP-32 (AP-32), prebiotic residual medium (RM) from AP-32 cultured medium, and their combination (A-RM) performed neuroprotective effects against dopaminergic neuron loss and motor dysfunction in a unilateral 6-OHDA-induced PD rat model. These supplementations increased mitochondrial activities and glycolysis which might increase energy metabolism in the brain and muscle, thus prevented dopaminergic neuron loss and muscle atrophy. Additionally, these supplementations modulated SCFAs production and increased antioxidative enzyme activities which might play important role in protecting mitochondria against reactive oxygen species (ROS). Supplementation of AP-32 showed the best performances among all supplementation groups. Therefore, AP-32 is the potential alternative as nutritional supplements in the treatment of PD. However, more detailed studies are required to investigate the mechanistic role of *Lactobacillus salivarius* subsp. *salicinius* AP-32 and prebiotic RM in attenuating PD progression.

## Data Availability Statement

The original contributions presented in the study are included in the article/Supplementary Material, further inquiries can be directed to the corresponding author/s.

## Ethics Statement

The animal study was reviewed and approved by Taipei Medical University Animal Care and Use Committee of Panel (IACUC/IACUP) (approval no. LAC-2020-0183).

## Author Contributions

H-YH designed and supervised the experiments. BAN, S-PT, and BP performed the experiments. C-HW and P-SH contributed reagents, materials, and analysis platforms. BAN and S-PT analyzed the data. BAN, S-PT, H-YH, and T-HY interpreted the results. S-PT prepared the figures. BAN and H-YH drafted the manuscript. All authors have read and agreed to the published version of the manuscript.

## Conflict of Interest

P-SH was employed by the company Bioflag Biotech Co., Ltd., Tainan City, Taiwan. The remaining authors declare that the research was conducted in the absence of any commercial or financial relationships that could be construed as a potential conflict of interest.
