# Supplementary material for: Probiotic Supplementation Facilitates Recovery of 6-OHDA-Induced Motor Deficit via Improving Mitochondrial Function and Energy Metabolism
Source: Front Aging Neurosci. 2021 May 7;13:668775. doi: 10.3389/fnagi.2021.668775 (PMC8137830; doi:10.3389/fnagi.2021.668775)
Supplement: Supplementary file 1 [file Table_1.DOCX]

Supplementary Material

Supplementary Table 1. Nutritional contents of residual medium (RM)

| Nutrients | Concentration (per 100 g) |
| --- | --- |
| Calories | 338.1 kcal |
| Crude fat | 2.1 g |
| Carbohydrate | 62.9 g |
| Crude protein | 16.9 g |
| Aspartic acid / Aspartate | 6.51 mM |
| Serine | 16.33 mM |
| Glutamic acid / Glutamate | 17.92 mM |
| Glycine | 20.58 mM |
| Histidine | 10.07 mM |
| Arginine | 19.38 mM |
| Threonine | 15.94 mM |
| Alanine | 54.31 mM |
| Proline | 3.66 mM |
| Cysteine | 0.00 mM |
| Tyrosine | 23.25 mM |
| Valine | 23.03 mM |
| Methionine | 8.03 mM |
| Lysine | 10.45 mM |
| Isoleucine | 16.12 mM |
| Leucine | 43.80 mM |
| Phenylalanine | 28.64 mM |
| Tryptophan | 8.07 mM |
| Total short-chain fatty acids | 9.2709 mM |
| Acetic acid | 7.0191 mM |
| Propionic acid | 0.1150 mM |
| Isobutyric acid | 0.0095 mM |
| Butyric acid | 0.0420 mM |
| Isovaleric acid | 0.0165 mM |
| Valeric acid | 0.0315 mM |
